# Editorial: Perspectives on Theory and Methods

**DOI:** 10.5334/pme.2812

**Published:** 2026-07-13

**Authors:** Anél Wiese, Terese Stenfors, Erik Driessen, Meredith Vanstone

**Affiliations:** 1 Medical Education Unit, School of Medicine, University College Cork, Cork, Ireland; 2 Department of Learning, Informatics, Management and Ethics, Karolinska Institutet, Stockholm, Sweden; 3 School of Health Professions Education, Faculty of Health Medicine and Life Sciences, Maastricht University, Maastricht, The Netherlands; 4 Department of Family Medicine, McMaster University, Hamilton, Canada

We are pleased to introduce a new section in Perspectives on Medical Education (PME): *Perspectives on Theory and Methods*. This editorial explains the rationale behind this section, what kinds of contributions we seek, and what we aim to achieve.

## Why this section?

Important conversations about theory and methods in health professions education (HPE) research happen frequently, often in a transient and ephemeral way that is not recorded or widely shared. A researcher asks in a conference hallway whether realist evaluation is appropriate for their implementation study. A supervisor explains to a PhD student why their sampling approach does not match their stated methodology. A reviewer writes detailed comments about why a common analytical technique does not actually do what the authors claim. These conversations are substantive, they advance methodological thinking, but they remain inaccessible to a larger audience.

The field of medical education does have resources on theory and methods. Journals publish methodological guidelines [1] including several in PME [2,3]. Special series explore specific approaches [4,5,6,7,8]. Methodological briefs appear occasionally [9]. One-page methodological features make concepts accessible to broad audiences, but cannot accommodate the detail researchers need to rigorously apply theory and methods in their own work [10]. This work is valuable, and it has advanced the field, but there is room for additional systematic assembly of this deep and thorough guidance on theory and methods. A researcher facing a challenge with theory or methods must hunt for guidance that may or may not exist and often finds silence where they need scholarly dialogue and advice.

Meanwhile, important advances in research theory and methods have no clear home within medical education. Researchers adapt methods from other fields to HPE contexts through trial and error rather than building on documented attempts. Common practices persist unchallenged because the critique exists only in private correspondence. Innovations get used once, briefly mentioned in a methods section, then lost.

How we approach theory and methods is not incidental to research quality and these advances should be recorded so they can be used. How we design studies, what methods we choose, how we analyse data – these decisions shape what we can learn about health professions education. When knowledge about theory and methods remains tacit, the field cannot systematically build capacity. A dedicated section on research theory and methods ensures sustained, cumulative conversation. PME’s commitment to open access means these resources reach researchers regardless of institutional subscriptions, which is critical for building global capacity.

## What kinds of contributions are we looking for?

We welcome papers that contribute to conversations about theory, methodology, and methods in HPE research. We use the terms theory, methodology, and methods deliberately, recognising these are contested distinctions in the literature [11]. For this section, we adopt the following working definitions:

**Theory papers** conceptualise educational phenomena. They explain why things happen or offer frameworks for understanding. For example, a paper demonstrating how Bourdieu’s concept of habitus illuminates professional identity formation in ways that cognitive theories cannot.

**Methodology papers** address research design and paradigms. They guide how to design studies that answer particular types of questions. For example, a paper explaining when case study methodology is appropriate for implementation research in medical education, including quality criteria and design principles.

**Methods papers** focus on specific data collection or analysis techniques. They provide practical guidance on executing particular procedures. For example, a paper detailing how to conduct think-aloud protocols in simulation settings, including facilitation and common pitfalls.

These boundaries are porous (a paper on realist evaluation might address all three), but these definitions help authors assess fit and help us communicate scope to reviewers.

Contributions can take several forms. Table 1 provides an overview of typical characteristics, though papers may combine elements. In the first contribution to the new section, Wiese, Maggio and Bennet argue that reflexive thematic analysis is not always methodologically appropriate for scoping reviews, and recommend alternative analysis methodologies [12]. Varpio argues in her commentary on the Wiese, Maggio and Bennet paper that researchers articulate the epistemological stance guiding a scoping review and ensure that the chosen analytic practices follow coherently from that stance [13].

**Table 1:** Contribution types for the Perspectives on Theory and Methods section.


TYPE	PURPOSE	EXAMPLE TOPICS

**Guidance and Innovation**	Introduce new tools or adaptations in an accessible way	How to conduct realist reviews in HPE; Rigorous approaches to member checking; Adapting participatory action research for virtual settings; Integrating AI-assisted analysis with qualitative rigour

**Critical Analysis and Debate**	Improve practice by addressing systemic issues and help researchers navigate contested areas	How “thematic analysis” is used inconsistently across HPE literature; Quality criteria in qualitative research: paradigm-specific or universal?; Common misapplications of data sufficiency

**Theoretical Contributions**	Apply theory to crucial issues and demonstrate utility	Practice theory for understanding workplace learning; When actor-network theory illuminates (and when it does not)


We are looking forward to the conversations this section will generate. The scholarship on theory and methods that HPE research needs is already happening. This section creates space to make that work visible and useful to the field. We invite you to be part of building this resource.
